# Complete association between a retroviral insertion in the tyrosinase gene and the recessive white mutation in chickens

**DOI:** 10.1186/1471-2164-7-19

**Published:** 2006-02-05

**Authors:** Chung-Ming Chang, Jean-Luc Coville, Gérard Coquerelle, David Gourichon, Ahmad Oulmouden, Michèle Tixier-Boichard

**Affiliations:** 1 UMR Génétique et Diversité Animales, INRA/INA P-G, Centre de Recherches de Jouy, 78352 Jouy-en-Josas, France; 2 Unité Expérimentale de Génétique Factorielle Avicole, INRA, Centre de Recherches de Tours, 37380 Nouzilly, France; 3 UMR Génétique Moléculaire Animale, INRA/Université de Limoges, 87061 Limoges, France

## Abstract

**Background:**

In chickens, three mutant alleles have been reported at the *C *locus, including the albino mutation, and the recessive white mutation, which is characterized by white plumage and pigmented eyes. The albino mutation was found to be a 6 bp deletion in the tyrosinase (*TYR*) gene. The present work describes an approach to identify the structural rearrangement in the *TYR *gene associated with the recessive white mutation.

**Results:**

Molecular analysis of the chicken *TYR *gene has revealed a major structural difference (Restriction Fragment Length Polymorphism, RFLP) in the genomic DNA of the recessive white chicken. A major size difference of 7.7 kb was found in intron 4 of the *TYR *gene by long-range PCR. Molecular cloning and sequencing results showed the insertion of a complete avian retroviral sequence of the Avian Leukosis Virus (*ALV*) family. Several aberrant transcripts of the tyrosinase gene were found in 10 week old recessive white chickens but not in the homozygous wild type colored chicken. We established a rapid genotyping diagnostic test based on the discovery of this retroviral insertion. It shows that all homozygous carriers of this insertion had a white plumage in various chicken strains. Furthermore, it was possible to distinguish heterozygous carriers from homozygous normal chickens in a segregating line.

**Conclusion:**

In this study, we conclude that the insertion of a complete avian retroviral sequence in intron 4 of the tyrosinase gene is diagnostic of the recessive white mutation in chickens. This insertion causes aberrant transcripts lacking exon 5, and we propose that this insertion is the causal mutation for the recessive white allele in the chicken.

## Background

**Figure 1:**
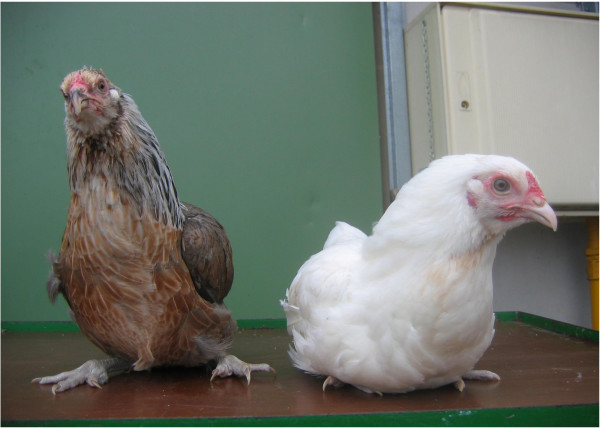
**Comparison of plumage color in full sib chickens differing for their genotype at the *C *locus**. On the left, a chicken carrying the wild type allele at the *C *locus exhibits a colored plumage as determined by other feather color loci. Here the animal carries the wild type allele at the Extension locus, the wild type allele at the Columbian locus and the silver allele at the Silver locus. On the right, a recessive white chicken, full sib from the previous one, exhibits full white plumage.

In birds and mammals, pigmentation of the feather and fur is determined mainly by the distribution of two melanin pigments, eumelanin (black-brown pigment) and phaeomelanin (yellow-red pigment). The synthesis of both pigments depends on tyrosinase, the key enzyme in melanin biogenesis in pigment cells, which catalyzes tyrosine in the first two biochemical steps resulting in the production of dihydroxyphenylalanine (DOPA) and dopaquinone [1]. Tyrosinase also catalyses the subsequent step in the formation of eumelanin [2] with the dehydrogenation of 5,6-dihydroxyindole-2-carbonic acid (DHICA). Without a proper enzymatic function of tyrosinase, the melanin synthesis pathway is blocked or incomplete; the animals exhibit an albino phenotype. In humans and mice, the *C *locus has been genetically defined as the structural tyrosinase gene. In chickens, three mutant alleles have been reported at the *C *locus in addition to the wild type allele (C*N), which is the most dominant allele with full pigmentation. These mutations are the red-eye white (C*RE), the recessive white (C*C) and the autosomal albino (C*A) [3]. They all give a white plumage but differ by pigmentation of the eye varying from a grey color to a totally non-pigmented albino phenotype [4,5] (Figure 1). Furthermore, day-old chicks may exhibit a lightly pigmented down at hatch in homozygous carriers of the C*C mutation. As reported previously [5], the recessive white (C*C) is one of the earliest traits to be studied in chicken genetics, applying Mendel's rules to segregating families for feather color patterns. The recessive white phenotype is a varietal characteristic of many breeds, such as the Plymouth Rock, Wyandotte, Minorca, Orpington, Jersey Giant, Dorking, Langshan, and Silky [5].

The chicken tyrosinase gene has been cloned [6] and its polymorphism has been characterized in the albino chicken (C*A/C*A) by Tobita-Teramoto *et al*. (2000) who reported a six nucleotide deletion in the tyrosinase coding sequence of the albino chicken [7]. So far, the molecular structure of the tyrosinase gene has not been studied for the two other alleles C*RE and C*C. In this study, we performed a molecular analysis of the tyrosinase gene in recessive white chickens in order to investigate the gene polymorphism and localize the causal mutation. We identified an avian retroviral sequence insertion in the tyrosinase gene of recessive white chickens in complete association with the mutant phenotype. Moreover, we established a rapid, sensitive and accurate diagnostic genotyping test that would be very helpful for breeders to identify heterozygous carriers of this recessive mutation.

## Results

### RFLP analysis

**Figure 2:**
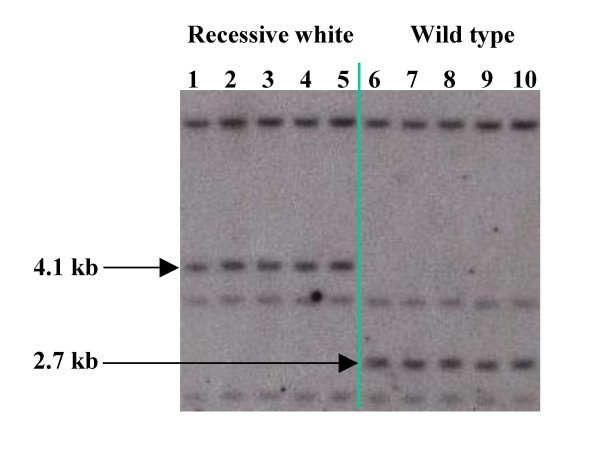
**RFLP banding pattern revealed by hybridization with a total chicken tyrosinase cDNA probe after H*in*dIII digestion of genomic DNA from recessive white mutant and wild type chickens**. Lane 1, 2, 3, 4, 5: recessive white chicken genomic DNA. Lane 6, 7, 8, 9, and 10: wild type (colored chicken) genomic DNA. The 4.1 kb band was only found in homozygous recessive white chickens while the 2.7 kb band was only found in the wild type chickens.

**Figure 3:**
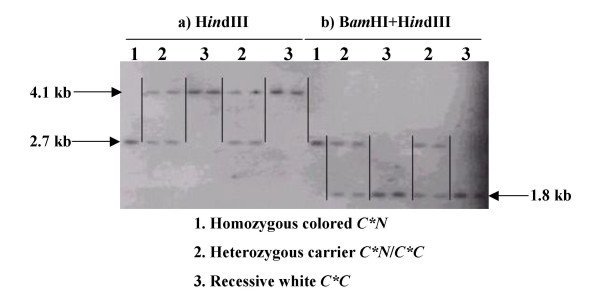
**RFLP banding pattern revealed by hybridization with a partial tyrosinase cDNA probe**. The partial cDNA probe included the total exon 5 region of chicken tyrosinase cDNA. This probe identified exactly the same restriction pattern as the total chicken tyrosinase cDNA probe. A simple H*ind*III digestion yielded 4.1 kb and 2.7 kb fragments in carriers of the recessive white or wild type allele at the *C *locus, respectively. A double digestion with B*am*HI+H*ind*III enzymes yielded a 2.7 kb fragment in chickens carrying the normal allele C*N and a 1.8 kb fragment in chickens carrying the recessive white allele C*C.

Four probes (Table 1) containing total or partial chicken tyrosinase cDNA, based on the sequence of White Leghorn chicken tyrosinase cDNA [6], were used. We observed a restriction fragment length polymorphism (RFLP) with 3 different enzymes [H*ind*III, Figure 2, (E*co*RI, B*am*HI data not shown)] in all recessive white mutant chickens using a total chicken tyrosinase cDNA probe. These results strongly suggest a major rearrangement in the structure of the *TYR *gene of the recessive white mutant (Figure 2). In order to more accurately localize this rearrangement, we used partial probes containing different coding regions of the chicken tyrosinase cDNA. Noticeably, probe TyrC which contained exon 5, revealed the same diagnostic bands between normal and mutant chickens as did the full cDNA probe. This result showed that the structural difference of the *TYR *gene between the recessive white mutant and the wild type chicken was located in the 3' terminal region of the *TYR *cDNA (Figure 3).

### PCR and DNA sequencing analysis

PCR amplification was performed for each of the five inferred exons of the chicken *TYR *gene (Table 2). All five exons were amplified from genomic DNA of recessive white chickens as well as wild type chickens. The results showed no size difference of amplified fragments between recessive white and wild type animals. Direct sequencing in both directions was performed to further investigate a possible sequence difference between carriers of the white mutation and colored chickens. The sequencing results of the five exons show that exon sequences were 100% identical for both genotypes. This result established that the rearrangement region of the *TYR *gene in the recessive white chicken was not in the coding sequence.

### Long-range PCR and intronic DNA sequencing

**Figure 4:**
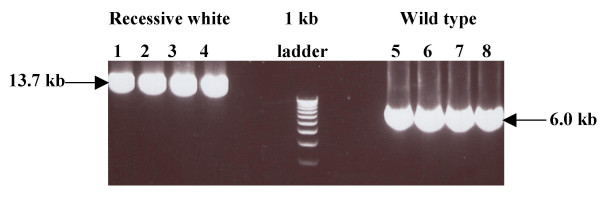
**Long-range PCR amplification of intron 4 in recessive white and wild type chickens**. The size of intron 4 of the *TYR *gene is 13.7 kb in the recessive white (left) and 6.0 kb in the wild type chicken (right).

**Figure 5:**
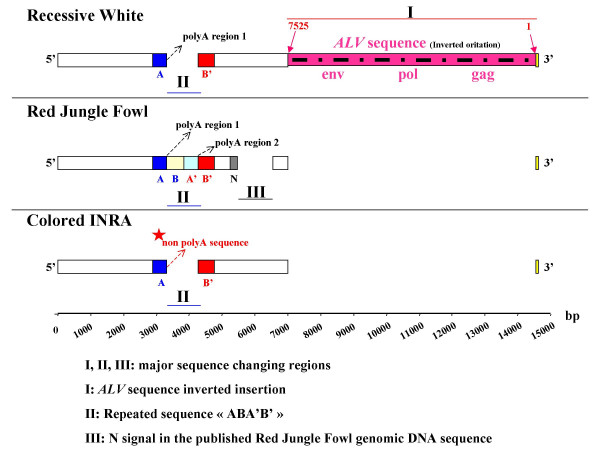
**Comparative analysis of intron 4 sequence of the *TYR *gene of a recessive white chicken, a colored chicken and the Red Jungle Fowl**. **I**: A complete *ALV *sequence was found in the recessive white chicken, inserted in the reverse orientation as compared to the usual *ALV *sequence numbered from 1 to 7525. **II**: A and A' or B and B' sequences respectively have up to 90% identity in the Red Jungle Fowl intron 4 sequence, and both A and A' sequences end by a polyA repeat (45 bp). Both of the recessive white and the wild type colored INRA chickens do not have B and A' sequences in their intron 4. The recessive white intron 4 "A" region contains the polyA sequence but not the "A" region of the wild type colored chicken intron 4. **III**: This region contains a stretch of N signals (chromosome 1: position 179546417–1795546627) in the published Red Jungle Fowl genome sequence that we were able to replace by a 1 kb informative sequence in both the recessive white and the wild type colored INRA chicken.

Once we had confirmed that the structural difference of the *TYR *gene in the recessive white chicken was near the 3' terminal region of the *TYR *cDNA and not in the exon sequence, a long-range PCR (Figure 4) procedure was performed in order to amplify intron 4 of the chicken *TYR *gene in both recessive white and colored chickens. A major size difference of 7.7 kb was observed in intron 4 between the recessive white mutant (13.7 kb) [GenBank:DQ118701] and the normal genotype (6.0 kb) [GenBank:DQ118702]. Further sequencing of the total intron region of a homozygous recessive white chicken revealed the presence of a 7.5 kb full-length retrovirus. This insertion was found in inverted orientation within intron 4 of the *TYR *gene and showed 99% (7458/7525) identity with the sequence of the avian leucosis endogenous virus *ev*-1 [GenBank:AY013303] [8] after using the BLASTn program [9]. The comparative analysis of the intron 4 sequence between the two genotypes (recessive white or colored chickens) and the published sequence for the Red Jungle Fowl [10] revealed three major sequence changes between the recessive white chicken and the Red Jungle Fowl and two major sequence changes between the colored INRA chicken and the Red Jungle Fowl (Figure 5). In addition to the retroviral insertion, intron 4 from the recessive white chicken exhibited a polyA stretch in position 3272 – 3310, which was not found in the colored chicken but was found in the Red Jungle Fowl which has a colored plumage also. Therefore, the insertion of the retroviral sequence in the *TYR *intron 4 sequence was the single critical change found in the recessive white chicken as compared to the colored chicken and the Red Jungle Fowl.

**Figure 6:**
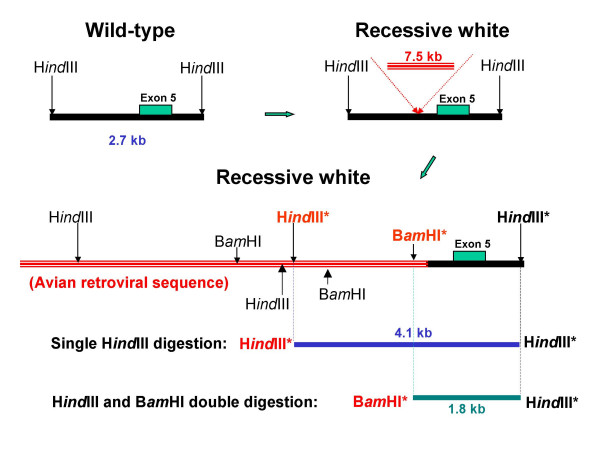
**Explanation of the RFLP results obtained for the *TYR *gene in recessive white chickens according to the structure of intron 4**. A 2.7 kb H*ind*III fragment was obtained in the wild type chicken after a single H*ind*III digestion and hybridization with an exon 5 probe. A 4.1 kb H*ind*III band and a 1.8 kb B*am*HI-H*ind*III band were obtained after H*ind*III single digestion and B*am*HI-H*ind*III double digestion in the recessive white mutation.

A comparison of sequence analysis with the chicken genome sequence [10] also provides a clear explanation for the previous RFLP results. The inserted avian retroviral sequence has added H*ind*III and B*am*HI restriction sites upstream of exon 5 of the chicken *TYR *gene, which results in a 4.1 kb H*ind*III band and a 1.8 kb B*am*HI-H*ind*III band after a H*ind*III single digestion or a B*am*HI-H*ind*III double digestion and hybridization with an exon 5 probe (Figure 6).

### Diagnostic genotyping test

**Figure 7:**
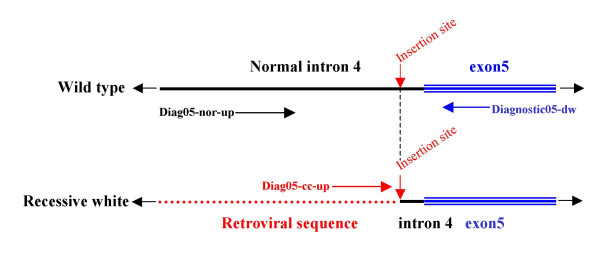
**Principle of the PCR diagnostic test for the molecular identification of the insertional mutation in intron 4 of the chicken *TYR *gene**. Two fragments may be obtained: 481 bp between Diag05-nor-up and Diagnostic05-dw for normal C*N allele, and 345 bp between Diag05-cc-up and Diagnostic05-dw for the C*C allele.

**Figure 8:**
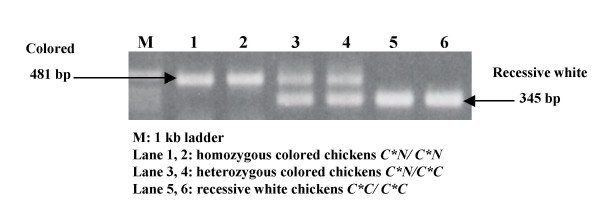
**Diagnostic genotyping test result**. Only one band either 481 bp or 345 bp was found in homozygous colored or homozygous recessive white chickens, respectively. In heterozygous chickens, both 481 bp and 345 bp bands were detected by the diagnostic genotyping test.

We designed three primers to establish a PCR diagnostic test after analyzing the intron 4 sequence of the colored and recessive white chicken (Figure 7). The homozygous colored chicken and homozygous recessive white chicken showed a single band of the expected size, either 481 bp or 345 bp respectively, while the heterozygous chicken showed both 481 bp and 345 bp bands after electrophoresis in agarose gel (Figure 8).

A total of 374 samples were tested from our experimental line (set 1) and from French slow-growing commercial lines (set 2); they included 115 chickens showing the recessive white phenotype, and 259 colored chickens (Table 3). According to the diagnostic test, all the 115 recessive white chickens from various origins were homozygous carriers of the retroviral sequence insertion and no colored chicken was found to be homozygous for the insertion. A total of 111 heterozygous carriers and 148 homozygous normal were found among colored chickens, which was consistent with the segregation of the recessive white mutation in the lines studied. The results obtained in the nine INRA sire families also confirmed that the retroviral sequence inserted in the *C *locus is stable and heritable in a Mendelian way; all the genotypes observed in the progeny were consistent with the parental genotypes.

Thus, the accuracy of the diagnostic test achieved 100% on 115 white recessive chickens from six different populations in France. This test is rapid, sensitive, and specific to distinguish the C*N/C*N homozygous, C*C/C*C homozygous and C*N/C*C heterozygous birds.

The last data set included 73 samples from 10 unrelated breeds and showed that the mutation was rare but could be found at various frequencies in a few populations that were exhibiting a white plumage phenotype (Table 4). The animals of the White Silky breed were homozygous C*C/C*C, which shows that this mutation did not prevent skin pigmentation. The mutation was not found in any of the population showing only colored animals. However, the recessive white mutation was not found in some white birds, which originated from breeds (Padova and White Leghorn) that are expected to carry the dominant white mutation. Regarding the broilers, the history of these lines mentions the presence of the Dominant White in the Cornish breed, an ancestor of broiler lines, as well as the presence of the recessive white in the Plymouth Rock and New Hampshire breeds, which were also used as ancestors of broiler lines. Thus, it is likely that current broilers may carry one or both of these mutations.

### 5' and 3' Rapid amplification of the cDNA end (RACE) of the TYR gene and sequence analysis

**Figure 9:**
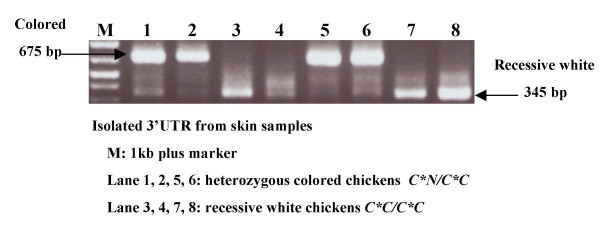
**Shorter 3'UTR are isolated by RACE from recessive white skin samples**. The major 3'UTR isolated in the recessive white was 345 bp and the major 3'UTR isolated in the heterozygous colored chicken was 675 bp.

**Figure 10:**
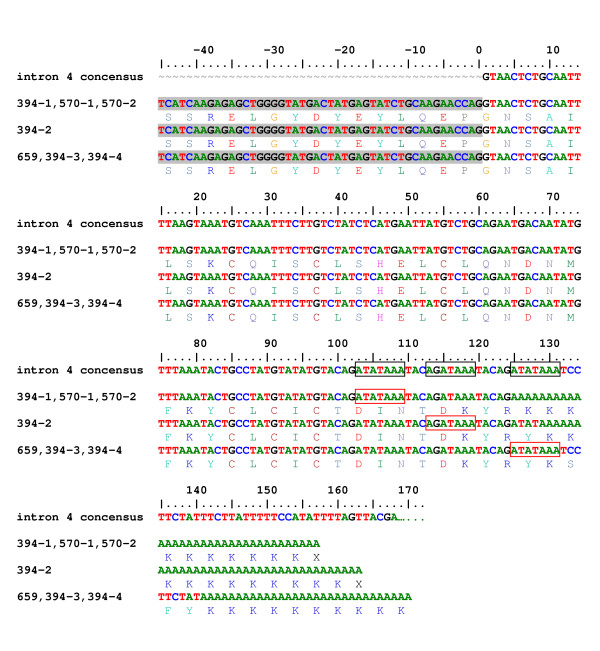
**Nucleotide and deduced amino acid sequences of 7 major transcripts belonging to 3 types of recessive white chickens that were isolated by RACE and compared with the published Tyrosinase cDNA sequence**. All of these 7 transcripts had normal exon 1 to exon 4 sequences but lacked the exon 5 sequence. A short sequence (from 124 bp to 140 bp) of intron 4 replaced the exon 5 sequence followed by a polyA tail. The exon 4 sequence is shown in grey color. Three putative polyadenylation signals ATATAAA, AGATAAA and ATATAAA in the intron 4 sequence are shown in boxes. For each transcript, the proposed polyadenylation signal is shown in the red box. The predicted amino acid sequence is shown below the nucleotide sequence.

The 5' and 3' end of the *TYR *gene cDNA were obtained by RACE (Table 2) from skin samples of 10 week old chickens, either colored and heterozygous carrier of the mutation, or homozygous for the recessive white allele. Isolated 5' and 3' end fragments, were first aligned with the exon sequences to complete the total mRNA transcript sequence. The complete transcript sequences were compared to the published chicken *TYR *cDNA sequences [6] and to the published Red Jungle Fowl sequence [10] by BLAST [9]. The 3' RACE result revealed that, in the skin of recessive white chickens, the 3'UTR was shorter than in colored chickens (Figure 9). These short transcripts did not contain exon 5. They exhibited a normal sequence from exon 1 to exon 4 and retained a short 5' flanking sequence from intron 4 (124 to 140 bp) before terminating with a polyA tail (Figure 10). There putative polyadenylation signals were found in the intron 4 sequence. By using different polyadenylation signals, the aberrant transcripts found in recessive white chickens may be classified in three types, of a different length, the consensus sequence of each type is shown on Figure 10. There was no sequence difference in the 5' end of the *TYR *gene mRNA amplified from skin samples of both colored and recessive white chickens.

### TYR gene expression tested by RT-PCR

**Figure 11:**
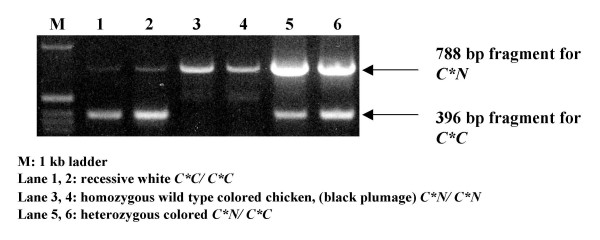
**RT-PCR detection of aberrant and normal-sized transcripts of the *TYR *gene**. The major transcript in the recessive white genotype was the truncated transcript (lane 1, 2) and the major transcript in the heterozygous colored chicken was the normal sized transcript (lane 5, 6). There was no aberrant transcript in the homozygous black plumage chickens (lane 3, 4).

A set of six birds (2 recessive white, 2 homozygous wild type colored and 2 heterozygous colored chickens) was tested by RT-PCR. RT-PCR results confirmed that the major transcript in the recessive white chicken is a truncated transcript but a very low level of expression of the normal transcript was detected (Figure 11). In contrast, there was only a normal-sized transcript in homozygote colored chickens. For the heterozygous colored chicken, the amount of the normal transcript was apparently higher than that of the aberrant transcript.

## Discussion

We report for the first time in chickens, as well as in vertebrates, a mutation of the tyrosinase gene that involves intron 4 and does not affect the coding sequence of any of the 5 exons. Indeed, the albino alleles described at the *TYR *locus are usually due to a single nucleotide substitution or a single nucleotide insertion/deletion in the coding sequence [11,12], but not in the intronic sequence. As an example, the dark-eyed albino mutation(*c*^*44H*^) in the mouse [13] presents a high phenotypic similarity with the recessive white chicken because it exhibits a white coat color, with pigmented eyes. This mutation was found to be due to a single base point mutation in exon 1 of the mouse tyrosinase gene, but no information was provided regarding the intron sequences. Another case of a white-coat animal with pigmented eyes was described in rabbits [14], and could not be associated to any mutation in the coding sequence. Furthermore, a screening of 120 cases of human oculocutaneous albinism (OCA) revealed the absence of any mutation in the coding sequence of the tyrosinase gene for 35 cases [15], 24 of these 35 were found in probands with the OCA1B condition, where minimal-to-moderate amounts of cutaneous and ocular pigment could be found. Our results show that it may not be possible to elucidate the mechanism of a tyrosinase mutation in some phenotypes showing white hairs or white coat and eye pigmentation, unless intron structure, and, if possible, intron sequence, are studied.

The retroviral insertion found in intron 4 of the tyrosinase gene may modify the gene's transcription pattern. Indeed, the 3'UTR results revealed that aberrant transcripts were generated in the recessive white chicken by alternative splicing and using three alternate polyadenylation sites that are present in the normal intron 4 sequence (Figure 10). It is most likely that the retroviral insertion plays a very important role in the splicing procedure and causes aberrant mRNA.

Tyrosinase exon 5 is the carboxyl terminal membrane spanning domain [16-18] which has an important role for the proper positioning of the enzyme in the melanosome [19]. A defect in the trafficking of the tyrosinase protein may affect the biogenesis of melanosomes [20] but melanosomal transfer to the keratinocytes is not well characterised. Disruption of this process would be expected to have severe consequences for pigmentation [21]. Misrouting of tyrosinase was observed in the platinum allele at the mouse albino locus, which is characterized by the occurrence of an abnormal stop codon in position 489 within exon 5 of the *TYR *gene [22]. This mutation is responsible for an extremely diluted coat color and pink eyes. In a human case, a single base insertion located in the transmembrane domain of the tyrosinase gene eliminated a portion of the transmembrane region and the carboxy terminus, and resulted in an inactive enzyme causing tyrosinase-negative oculocutaneous albinism [23]. In the case of the chicken recessive white mutation, the lack of exon 5 in the transcript could affect the translation of the membrane spanning domain although the conserved copper-binding regions are retained; this transcript may therefore encode a cytoplasmic, rather than a membrane-bound enzyme, and this would disturb melanogenesis. Although the truncated transcripts do not have a stop codon before the polyadenylation, they are still the major transcripts in the recessive white chicken. Previous studies of tyrosinase in the mouse suggested that shorter cDNA resulting from alternative splicing may have non functional tyrosinase activity [19]. Translation of the recessive white chicken alternative transcripts would be expected to result in a truncated protein. A previous study of the tyrosinase enzyme suggested that both C*C (recessive white) and C*A (albino) alleles could produce tyrosinase-like molecules that are inactive due to a change that is electrophoretically and antigenically "silent" [24].

The difference observed between the eye and the feather pigmentation will need further consideration. The retinal pigment epithelium (RPE) derives from the optic cup [25] and arises from different precursor cells than the skin melanocytes. Furthermore, the eye pigment cells do not transfer their pigment [26]. Thus, the consequences of a defect in pigment synthesis may be expected to differ in the eye and in the feathers. For instance, the dominant white mutation of the chicken has been found to be a defect of the *PMEL17 *gene [27] which codes for a membrane protein of the melanosome, this mutation suppresses black pigment from the feathers but does not affect eye pigmentation. The fact that some pigment appears in the eyes of recessive white chickens indicates some tyrosinase activity. In the case of the mouse dark-eyed albino mutation (*c*^*44H*^), the *TYR *gene expression was not affected by the point mutation, but the phenotypic consequences of this mutation were less severe in the eyes than in the coat. If a partially truncated tyrosinase protein is produced in the recessive white chicken, this may cause disorganization of pigment into melanosomes and may lead to an improper transfer to the feather keratinocyte, but it might not affect skin pigmentation, and could explain why the White Silky exhibits black skin with white plumage in the presence of a mutated tyrosinase gene. An alternative interpretation of the mechanism of the recessive white mutation is that the aberrant transcript does not give any functional protein at all, but that pigmentation is maintained in some tissues since a small proportion of the normal transcript is still produced in the mutant (see Figure 11). Maybe this is a sufficient amount in some tissues (especially in eyes) but not in the growing feather. The splicing may also be different according to tissues.

Retroviral insertions are known to be responsible for insertional mutagenesis in mice, as illustrated by the 'dilute' coat color mutation [28] and the hairless mutation [29]. In chickens, the henny-feathering mutation (Hf) results from an abnormal expression pattern of the aromatase gene, under the control of a retroviral long terminal repeat [30]. The sex-linked late-feathering mutation, K, is associated with the insertion of a full-size retroviral sequence, named *ALVE*21, together with the duplication of the insertion site [31]. Both Hf and K are dominant mutations. The K mutation exhibits some peculiar features due to its association with *ALVE*21: (i) revertants have been observed which carry a solitary long terminal repeat (LTR) in place of the full retroviral element [32], (ii) carriers of the K mutation exhibited a higher susceptibility to exogenous leucosis infection in some chicken strains such as White Leghorns, this phenomenon could be partly explained by the production of a full viral particle due to the expression of the *ALVE*21 genome. No such observation has been made for the recessive white mutation, which could suggest that the insertion found within intron 4 is quite stable and that the retroviral genome is not expressed or not sufficient to induce an interaction with exogenous retroviruses. Since the recessive white mutation has never been described as unstable, it may be questioned whether the insertion in inverted orientation may limit the occurrence of reversion events. Revertants could have been detected in our diagnostic test if only a solitary LTR was remaining at the site of insertion, then a larger band would have been amplified between the Diag05-nor-up and Diagnostic05-dw primers in some colored chickens, but this was not observed.

## Conclusion

The structural change (RFLP) initially identified in the *TYR *gene of recessive white chickens has been shown to result from the insertion of a complete avian retroviral sequence of 7.7 kb in intron 4 of the gene. The development of a rapid PCR diagnostic test made it possible to study the distribution of this insertion among various chicken populations, which showed that homozygous carriers were always found to have a white plumage, whatever the origin of the line. The finding of the same insertion in populations sharing the recessive white phenotype but as distantly related as a French experimental line derived from the Gâtinaise breed, a commercial slow-growing broiler line, and the Asiatic Silky breed, strongly supports that this retroviral insertion is not a spurious association with the recessive white mutation and could be the causal mutation. Indeed, truncated transcripts lacking exon 5 were identified in the skin of recessive white chickens, but were not found in the skin of colored chickens, homozygous for the normal allele. This represents a new model for the investigation of the effects of an intronic mutation in the *TYR *gene on the phenotype-genotype relationship, since all the mutations previously described for this gene across species affected the coding sequence.

Finally, a rapid diagnostic genotyping test is now available to breeders, in order to identify heterozygous carriers of the mutation, which could be otherwise identified only by a tedious progeny-test.

## Methods

### Animals

The recessive white mutation is segregating in a chicken line maintained at the INRA experimental farm 'UE-GFA' located in Nouzilly, near Tours. This mutation originated from a French traditional breed the "Gâtinaise" which was first characterized in 1953 [33]. For the present study, mating pairs were made between a heterozygous sire and either a homozygous mutant dam or a heterozygous dam, in order to produce progeny of both phenotypes, either "colored" or "recessive white". Within this line, a colored phenotype and a recessive white parent characterize a heterozygous animal. A first set of three full-sib families with a total of 11 offspring, 5 recessive white and 6 colored, was used for RFLP, PCR, 5' and 3' RACE and sequencing studies. In addition, six homozygous normal animals were sampled in unrelated families of another experimental line in order to obtain reference animals with a fully colored genotype; one of these was used for sequencing of the *TYR *gene.

Another sampling procedure was set up in order to confirm the association between the retroviral insertion and the recessive white phenotype. It involved a first set of 145 animals from our recessive white experimental line, and a second set of 229 chickens from five commercial lines of slow-growing type chickens used for 'Label Rouge' production or certified production (Table 3). These commercial lines were of different origin and one of them (line A) was homozygous for the mutation, and had been imported by a private breeder into France. A third set of 73 chickens was sampled in 10 chicken breeds with various color phenotypes (Table 4). Most of these breeds had been studied in the framework of the AvianDiv European research project [34] and the White Silky breed was sampled in our INRA experimental facilities. This is the white feathered variety of the Silky breed which shows black skin. The special trait of hypermelanic pigmentation in the Silky breed is due to dermal melanin and is independent of feather color, since several varieties of this breed are described with black skin and variable plumage color, either white, black, or gold [35].

### DNA extraction

Blood samples for genomic DNA extraction were prepared from 10 week old recessive white and colored chickens. High molecular weight genomic DNA was extracted from 80 μl blood after hemolysis at 4°C followed by incubation with 200 μg/mL proteinase K, precipitation with 4.5 mL dimethylformamide/acetone (5:95 vol/vol), resuspension into TE buffer, and a second precipitation with 100% ethanol. Genomic DNA was finally resuspended in 2 mL of TE buffer and its concentration was determined by spectrophotometer [36]. Crude DNA extractions were prepared by incubating 2 μl of whole blood with 250 μl of NaOH (0.2 M) at 65°C for at least 2 h followed by neutralization with 250 μl of Tris-HCl (0.2 M). These crude extracts were used for the PCR genotyping test.

### RFLP and southern analysis

Eight micrograms of genomic DNA were digested overnight at 37°C with a single restriction enzyme B*am*HI, E*co*RI or H*ind*III (5 units/μg) or with double digestion using B*am*HI and H*ind*III. All digested DNA samples were fractionated on a 0.8% agarose gel by electrophoresis at 2 V/cm for 22 h, and blotted onto a charged nylon membrane in 0.4 M NaOH. Four probes were labeled with digoxigenin and the random-priming DNA labeling kit 'High-Prime kit' (Roche diagnostics, Meylan, France). Hybridization was performed at 42°C according to the standard Roche Applied Science procedure for digoxigenin-labeled probes. After a stringent membrane wash (0.5 SSC at 65°C), hybridized fragments were revealed by incubation with antidigoxigenin antibody conjugated with alkaline phosphatase which further reacts with Lumigen (Roche diagnostics, Meylan, France). Light emission was revealed with Kodak^® ^Biomax film after exposure at room temperature for 30 min to 20 h.

### PCR and DNA sequencing

The five exons of the chicken *TYR *gene were inferred from the coding sequence of the human *TYR *gene. Five pairs of primers were designed (Table 2) to separately amplify exons on genomic DNA in both chicken genotypes. After PCR amplification, DNA fragments were isolated, purified and sequenced by direct sequencing.

### Long-range PCR, molecular cloning and DNA sequencing

The Expand Long-template PCR procedure (Roche diagnostics, Meylan, France) was performed in order to study the structure of intron 4 of the chicken *TYR *gene in recessive white mutant chickens. Only purified genomic DNA was used for the long PCR reaction. The entire intron 4 of the chicken *TYR *gene of both genotypes (colored and recessive white) was amplified according to the manufacturer's instructions. The primers 5'-GCT GGG GTA TGA CTA TGA GT-3' and 5'-CTT GCT TGA GGT AGG GGA T-3' were at the 3' end of exon 4 and at the 5' end of exon 5 of the chicken *TYR *gene respectively. The long-range PCR amplification was performed in volumes of 50 μl containing 1 μl of each primer using 30 cycles under the following conditions: initial denaturation at 94°C for 2 min, 94°C for 10 sec (10 cycles) or 15 sec (20 cycles), 58°C for 30 sec, 68°C for 7 min, with a 20 sec additional extension for the last 20 cycles, followed by a final stage at 68°C for 7 min. After this amplification, six H*in*dIII fragments of intron 4 of the recessive white chicken were cloned into the pUC19 plasmid (Qbiogene, France). Two of the intronic H*in*dIII fragments (3.2 kb and 3 kb) of the recessive white chicken were sent to be sequenced by the MWG Biotech Company (Germany) under the Publication Quality Sequencing project. The entire intron 4 was also direct sequenced for both the recessive white and the wild type genotypes, and the other 4 intronic H*in*dIII fragments were also direct sequenced in both directions in the UMR1061 laboratory, on an ABI Prism 310 DNA Genetic Analyzer (Perkin-Elmer France). We used Sequencher 4.1 computer software (Gene Codes Corporation, Ann Arbor, Mich.) for sequence alignment. There were a few regions that were difficult to sequence directly from the long PCR amplification fragments, 3 regions in the recessive white and 2 in the wild type intron 4. These 5 regions were subcloned into the pCR2.1 vector (Invitrogen, France) and sequenced in both directions.

### Diagnostic genotyping test

The PCR reaction was performed with a mix of three primers: an upstream primer Diag05-cc-up 5'-CCT CTG GCT CTA TTT GAC TAC ACA GT-3' was located in the gag region of the retroviral sequence, and an upstream primer Diag05-nor-up 5'-CAA AAC CAT AAA TAG CAC TGG AAA TAG-3' was located in the normal sequence of intron 4, the downstream primer Diagnostic05-dw 5'-TTG AGA TAC TGG AGG TCT TTA GAA ATG-3' was located in exon 5 of the *TYR *gene. The PCR amplifications were carried out in a 25 μl reaction volume containg 10 pmol of each primer in the following cycling condition: initial denaturation 95°C for 3 min, followed by 35 cycles (95°C for 30 sec, 58°C for 30 sec, and 72°C for 1 min), and one cycle (72°C for 5 min). Two fragments were expected: 481 bp between Diag05-nor-up and Diagnostic05-dw, and 345 bp between Diag05-cc-up and Diagnostic05-dw.

The association between the insertional mutation in intron 4, detected by the 345 bp band in our test, and the recessive white phenotype was tested on a total number of 454 samples, from three independent sets of chicken lines.

### 5' and 3' Rapid amplification of cDNA end (RACE)

In order to analyse the 5' and 3' untranslated regions (UTR) of the *TYR *gene, RACE experiments were performed on 1 μg total RNA extracted from skin samples, using the SMART™ RACE cDNA Amplification kit (Ozyme, France), according to the manufacturer's instructions. 5' and 3' UTR of *TYR *gene transcripts were amplified by nested PCR with specific and adaptor primers: PL1/UPM (Universal Primer Mix) and PL2/NUP (Nested Universal Primer) for the first and second amplifications of 5'UTR respectively. P3'-1/UPM and P3'-2/NUP were used for the first and second amplifications of 3'UTR respectively. First and second PCR amplifications were carried out in a 25 μl reaction volume containing 10 pmol of each primer and 12.5 μl of 2× working concentration PCR Master Mix (ABgene France) in the following cycling conditions: initial denaturation at 95°C for 2 min followed by 35 cycles (95°C for 30 s, 61°C for 30 s, 72°C for 1 min) and one cycle (72°C for 5 min). PCR products from 5' and 3' RACE secondary PCR reaction were cloned into the pCR2.1 vector (Invitrogen SARL) and sequenced (ABI Prism 310 DNA Genetic Analyzer, Perkin-Elmer France).

### Study of the TYR gene expression by RT-PCR

Three primers were designed for the RT-PCR (Table 2). Primer pair exon3-up and exon5-dw (788 bp) amplifies the transcript region from exon 3 to its 3'UTR which is expected for the normal allele C*N. Primer pair exon3-up and 3' UTR-RE (396 bp) amplifies the truncated transcript which is expected for the recessive white allele C*C from exon 3 to the 3'UTR. RT-PCR amplification conditions were as follows: 96°C for 3 min, followed by 40 cycles of amplification (94°C for 30 s, 56°C for 30 s, 72°Cfor 1 min) and one cycle (72°C for 7 min).

## Authors' contributions

CMC carried out all experimental work described in the paper and drafted the manuscript. JLC realised the first RFLP studies and contributed to the molecular genetic studies. GC and DG participated in the design of the study and carried out the animal selection and sampling. AO participated in the design of the study and offered the technical supports of the 5' and 3' RACE amplification and sequencing study. MTB conceived and supervised the study, coordinated the project and finalized the manuscript. All authors read and approved the final manuscript.

## Figures and Tables

**Table 1:** Complete and partial *TYR *cDNA probes used in the RFLP analysis

*Probe name*	*First nucleotide*	*Last nucleotide*	*Total length (bp)*
Total *TYR *cDNA	1	1997	1997
TyrA	125	909	785
TyrB	930	1459	530
TyrC*	1501	1943	443

*Probe TyrC contains exon 5 and 3'UTR; exon 5: 195 bp; 3'UTR: 248 bp

Sequence Accession Number GenBank:D88349

**Table 2:** Description of the primers used in this study to amplify the five exons of the chicken *TYR *gene, for the RACE reaction and the RT-PCR

***Primers***	***Sequence 5' to 3'***	***Description***
exon1-up	TGGGCTTACTGCTGGTCATCC	Forward exon1
exon1-dwn	TGATGCTGGGCTGAGTAAATTAG	Reverse exon1
exon2-up	AATCTGCACTCAATCTGAAGAG	Forward exon2
exon2-dwn	TTCGGAAGCTGTAATTGGC	Reverse exon2
exon3-up	CCACACACTGCAATATCAAAC	Forward exon3
exon3-dwn	TCAACAAATGCATGGTGTAGG	Reverse exon3
exon4-up	CATTTTTGAGCGGTGGTTAAG	Forward exon4
exon4-dwn	ACTCATAGTCATACCCCAGC	Reverse exon4
exon5-up	ATCCCCTACCTCAAGCAAG	Forward exon5
exon5-dwn	CAGGAGTGTTGTTTAATCAAGC	Reverse exon5
PL1	ATGCGCTGGCAGGTTCCTCTGTTGGAACGC	5' RACE PCR1
PL2	CAGCACTCCTTCCTCAGCAAGCTCTGCGTG	5' RACE PCR2
P3'-1	AGCGGTGGTTAAGAAGACACAGACC	3' RACE PCR1
P3'-2	GCTAGAAGTTTACCCAGCAGCCAAC	3' RACE PCR2

3' UTR-RE*	AAACATATTGTCATTCTGCAGACA	3'UTR primer sequence for the C*C

Sequence Accession Number GenBank:D88349

3' UTR-RE* sequence is in the aberrant transcript 3'UTR region of the recessive white chicken tyrosinase gene

**Table 3:** The results of the PCR diagnostic test for intron 4 of the *TYR *gene on chickens sampled in the recessive white experimental line at INRA and in five commercial lines of slow-growing type chickens

**Set 1. Recessive white, experimental line, 145 birds**		**Phenotype**	**Size and number of amplified band(s) with the PCR diagnostic test**		
	Number		**345 bp**	**481 bp**	**481 bp+345 bp**

Sires	6	recessive white	**6**	**-**	**-**
	3	colored	**-**	**1**	**2**
Dams	3	recessive white	**3**	**-**	**-**
	41	colored	**-**	**5**	**36**
Progeny	38	recessive white	**38**	**-**	**-**
	54	colored	**-**	**4**	**50**

**Set 2. Commercial lines, slow-growing type, 229 birds**		**Phenotype**	Size and number of amplified band(s) with the PCR diagnostic test		

	Number		**345 bp**	**481 bp**	**481 bp+345 bp**

Line A	61	recessive white	**61**	**-**	**-**
Line B	30	colored	**-**	**30**	**-**
Line C	55	colored	**-**	**45**	**10**
Line D	5	recessive white	**5**	**-**	**-**
	26	colored	**-**	**15**	**11**
Line E	2	recessive white	**2**	**-**	**-**
	50	colored	**-**	**48**	**2**

**Table 4:** The results of the PCR diagnostic test on unrelated chicken breeds: the 345 bp corresponds to the mutant allele of the *TYR *gene and the 481 bp corresponds to the wild type allele

Breed (origin of samples)	**Phenotype**	Size and number of amplified band(s) with the PCR diagnostic test			**Total**
		**345 bp**	**481 bp**	**481 bp + 345 bp**	

Friesian fowl (AvianDiv)	Variable, always colored	**-**	**8**	**-**	**8**
Padova (AvianDiv)	Variable, including some white	**-**	**8**	**-**	**8**
Green-legged Partridge (AvianDiv)	Wild type, always colored	**-**	**8**	**-**	**8**
Ukrainian Bearded (AvianDiv)	Variable, including some white	**-**	**7**	**1**	**8**
Godollo Nhx (AvianDiv)	Colored (red)	**-**	**8**	**-**	**8**
White Leghorn (AvianDiv)	Full white	**-**	**7**	**-**	**7**
Rhode Islad Red (AvianDiv)	Colored (red)	**-**	**8**	**-**	**8**
Broiler sire line (AvianDiv)	Full white	**2**	**6**	**-**	**8**
Broiler dam line (AvianDiv)	Full white	**-**	**3**	**4**	**7**
Silky breed (INRA)	Full white with black skin	**3**	**-**	**-**	**3**
